# Characterization of the Skin Microbiota in Italian Stream Frogs (*Rana italica*) Infected and Uninfected by a Cutaneous Parasitic Disease

**DOI:** 10.1264/jsme2.ME15041

**Published:** 2015-09-15

**Authors:** Ermanno Federici, Roberta Rossi, Laura Fidati, Romina Paracucchi, Silvia Scargetta, Elena Montalbani, Andrea Franzetti, Gianandrea La Porta, Anna Fagotti, Francesca Simonceli, Giovanni Cenci, Ines Di Rosa

**Affiliations:** 1 Department of Chemistry, Biology and Biotechnology, University of Perugia, Via del Giochetto, 06122 Perugia, Italy; 2 Department of Earth and Environmental Sciences, University of Milano-Bicocca, Piazza della Scienza 1, 20126 Milan, Italy

**Keywords:** amphibian, skin microbiota, Illumina sequencing, *Amphibiocystidium*, *Rana italica*

## Abstract

In human and wildlife populations, the natural microbiota plays an important role in health maintenance and the prevention of emerging infectious diseases. In amphibians, infectious diseases have been closely associated with population decline and extinction worldwide. Skin symbiont communities have been suggested as one of the factors driving the different susceptibilities of amphibians to diseases. The activity of the skin microbiota of amphibians against fungal pathogens, such as *Batrachochytrium dendrobatidis*, has been examined extensively, whereas its protective role towards the cutaneous infectious diseases caused by *Amphibiocystidium* parasites has not yet been elucidated in detail. In the present study, we investigated, for the first time, the cutaneous microbiota of the Italian stream frog (*Rana italica*) and characterized the microbial assemblages of frogs uninfected and infected by *Amphibiocystidium* using the Illumina next-generation sequencing of 16S rRNA gene fragments. A total of 629 different OTUs belonging to 16 different phyla were detected. Bacterial populations shared by all individuals represented only one fifth of all OTUs and were dominated by a small number of OTUs. Statistical analyses based on Bray-Curtis distances showed that uninfected and infected specimens had distinct cutaneous bacterial community structures. Phylotypes belonging to the genera *Janthinobacterium*, *Pseudomonas*, and *Flavobacterium* were more abundant, and sometimes almost exclusively present, in uninfected than in infected specimens. These bacterial populations, known to exhibit antifungal activity in amphibians, may also play a role in protection against cutaneous infectious diseases caused by *Amphibiocystidium* parasites.

It is commonly accepted that plants and animals harbor assemblages of microbes and that such symbiotic microorganisms play crucial roles for their hosts (4, 40). The importance of natural microbiota in health maintenance and the prevention of diseases has been demonstrated in several systems (7, 15, 18, 21, 28, 52). Interactions between the host, symbiotic microbes, and pathogens have been shown to affect disease outcomes (28), and this represents a matter of special concern because emerging infectious diseases are increasingly threatening human and wildlife populations (32, 33).

The skin is the soft outer covering of vertebrates that interfaces with the environment. It provides the first line of defense from external factors, such as pathogens and harmful substances, prevents excessive water loss, and guarantees insulation and sensation. In amphibians, skin is a particularly complex organ as it plays key roles in respiration, osmoregulation, thermoregulation, pigmentation, chemical communication, and pathogen defense (10). Amphibian skin, owing to the presence of a glycoprotein-rich mucous layer, harbors a rich microbial community and, thus, represents a complex ecosystem shaped by interactions with the environment and host factors that influence colonization (48). Amphibians synthesize species-specific oligosaccharides that, together with components of the innate and adaptive immune systems, such as lysozyme, antimicrobial peptides, and mucosal antibodies, mediate the colonization process by specific microbial populations from the host’s environment (48).

Amphibians are faced with decline globally and emerging infectious diseases have been closely associated with this decline and, ultimately, extinction (5). Susceptibility to diseases may vary among individuals, populations, or species of amphibians, and their well-developed skin symbiotic communities have been suggested as one of the driving factors for these differences (4, 33, 41). The activity of amphibian cutaneous bacteria against the skin pathogen *Batrachochytrium dendrobatidis* (*Bd*) has attracted a lot of attention (2, 28–30). This fungus causes chytridiomycosis, an emerging infectious disease thought to be responsible for amphibian decline and extinction worldwide (22). On the other hand, some *Bd*-naive populations of amphibians have the ability to persist and co-exist with *Bd*, and population survival has been suggested to depend on the proportion of individuals with anti-*Bd* bacteria (24, 36, 56). Many factors have been suggested to play a role in amphibian disease resistance including host genetics, immunology, skin peptides, and environmental factors (48). Furthermore, bacteria isolated from amphibian skin are known to produce anti-fungal molecules that inhibit the growth of *Bd* (28), and have even been shown to reduce amphibian mortality under controlled experimental conditions (30).

Besides chytridiomycosis, amphibians are potentially threatened by other cutaneous diseases, among which include infections by parasites of the genus *Amphibiocystidium* in the class *Mesomycetozoea*, a group of microorganisms at the boundary between animals and fungi (42, 51). Such infections are macroscopically detectable by the presence of swellings in the skin on any part of the body (6, 26, 47) and may be associated with local declines in frog populations (17). In 2007, an infection by parasites of the genus *Amphibiocystidium* was detected for the first time in a population of the Italian stream frog (*Rana italica*) in Central Italy and is still occurring (49). *R. italica* is a stream-breeding anuran endemic of the Peninsular Italy, mainly occurring in forested areas associated with shady streams with a rocky substrate along the Apennine chain and adjacent hilly ranges (11), and is regarded as a species in need of strict protection by the European Union (Annex IV in the Council Directive 92/43/EEC).

A more detailed characterization of the microbial community structure and dynamics is needed in order to gain a better understanding of the relationship between the composition of the skin microbiota and health status. As in other environments, culture-based approaches are able to capture only a small fraction of the bacterial diversity present on amphibian skin (39). In this respect, next-generation sequencing technologies are advantageous because they provide a thorough description of microbial communities, including uncultivable members, due to high-throughput 16S rRNA gene tag-deep sequencing (25). Although this approach has been increasingly applied in recent studies to investigate skin-associated microbial communities in amphibians (3, 23, 35, 38, 41, 53), to the best of our knowledge, it has never been used to directly address bacterial populations with a potential defensive role against a cutaneous infectious disease, such as the infection by parasites of the genus *Amphibiocystidium*. Members of this genus, as well as other mesomycetozoeans, are uncultivable (42) and, thus, it is not possible to perform *in vitro* experiments in order to explore the inhibitory activity of cutaneous bacteria against *Amphibiocystidium*. The aim of the present study was to obtain information on the composition of the skin microbiota in the Italian stream frog (*R. italica*) using the Illumina next-generation sequencing of 16S rRNA gene fragments, and also to investigate differences in microbial assemblages between frogs infected and uninfected by *Amphibiocystidium* in order to infer the potential role of skin-associated bacteria in the defense against this parasite.

## Materials and Methods

### Sample collection

*R. italica* specimens were collected in April 2010 from a population in the Nestore Valley (Central Italy). The capture and handling of *R. italica* specimens were conducted according to the permission granted by the Italian Ministry of Environment (issue no. DPN-2009-0011282). Frogs were captured by hand using fresh latex gloves each time. Before swabbing, each frog was rinsed twice with sterile distilled water in order to remove transient bacteria not associated with the skin (37). In each specimen, sterile cotton-tipped swabs were drawn across the skin of the lateral, ventral, and dorsal surfaces of the body, thighs, and feet ten times for each site. Each swab was recovered into a sterile vial and stored at −20°C for later analyses. Frogs infected by parasites of the genus *Amphibiocystidium* were first identified by the presence of gross swellings on the skin and then confirmed by a histopathological examination following a previously reported method (45, 46). Six specimens, three uninfected (indicated as R151S, R155S, and R156S) and three infected (indicated as R150M, R152M, and R154M), were selected for a next generation sequencing analysis of the skin microbiota. All chosen frogs were females, with the only exception of R154M, which was a male. Specimen weights ranged from 6.2 to 9.4 g (average 8.2 g), while snout-vent lengths ranged from 40.25 to 45.09 mm (average 42.07 mm).

### Metagenomic DNA extraction and 16S rRNA gene fragment sequencing

Metagenomic DNA was extracted from each swab using the PowerSoil DNA isolation kit (MoBio Laboratories, Carlsbad, CA, USA). Swabs were loaded directly into the bead tubes provided and the remaining extraction procedure was performed as previously reported (20).

The V5-V6 hypervariable regions of the 16S rRNA gene were amplified in 50-μL volume reactions using the 5× HOT FIREPol Blend Master Mix (Solis BioDyne, Tartu, Estonia) with 1 μM each of the primers 783F and 1027R (25). A 6-bp barcode was also included at the 5′ end of the 783F primer to allow sample pooling and subsequent sequence sorting. Cycling conditions included initial denaturation at 94°C for 5 min followed by 29 cycles of 94°C for 50 s, 47°C for 30 s, and 72°C for 30 s and a final extension at 72°C for 5 min. The amplified products were purified with the Wizard SV PCR purification kit (Promega Corporation, Madison, WI, USA) and DNA quantity and purity were spectrophotometrically evaluated by NanoDrop (Thermo Scientific, USA).

Purified amplicons with different barcodes were pooled in 100-μL samples with a DNA concentration of 40 ng μL^–1^. Multiplexed sequencing of all the pooled samples was performed on a single Illumina Hiseq 1000 lane, using a paired-end 2×100 base-pair protocol and 4.0 sequencing chemistry. Cluster extraction and base-calling processing analyses were performed using the Illumina CASAVA Analysis software, version 1.8. Illumina Hiseq 1000 sequencing was carried out at BMR Genomics, Padua, Italy. The entire sequence dataset is available in the European Nucleotide Archive of the EMBL database with study accession number PRJEB7174.

Each sequence was assigned to its original sample according to its barcode. A quality cut-off was then applied in order to remove sequences i) that did not contain the barcode, ii) with an average base quality value (Q) lower than 30. The barcode was removed from sequences before further processing. The reverse read of each paired-end sequence was reverse-complemented and merged with the corresponding forward read, inserting 10 Ns in between (12). The number of reads recovered from each sample ranged from 612,786 to 1,016,572, while 10,000 reads per sample were randomly selected to normalize further analyses on the bacterial community structure (25).

The clustering of operational taxonomic units (OTUs) was carried out on the forward reads that contained the entire V6 region using the UPARSE-OTU algorithm (19). The minimum identity between each OTU member sequence and the representative sequence (*i.e.* the sequence that showed the minimum distance to all other sequences in the OTU) was set to 97%. OTUs that comprised only one read were removed. The taxonomic classification of the OTUs was carried out with the RDP classifier (54) using the representative sequence for each OTU.

Irrespective of their OTU assignment, all the filtered and merged reads were also given a taxonomic attribution using the stand-alone version of RDP Bayesian Classifier, applying 50% of confidence as suggested for sequences shorter than 200 bp (12).

### Statistical analysis

A cluster analysis of OTUs was carried out using the heatmap function in the R statistical environment. Heatmap is a color-shaded matrix that is useful for a quick overview or exploratory analysis of data, in which color intensity indicates the relative value of the occurrence of the OTU (a light color indicates a low value, while a dark color indicates a high value). A hierarchical plot was produced using an average linkage cluster analysis based on the Bray-Curtis dissimilarity matrix.

An analysis of similarity (ANOSIM) (13) was performed for comparisons of similarity between infected and uninfected groups. ANOSIM is a non-parametric, permutation-based test for significant differences in compositions or variations between groups, and returns a test statistic, R, with a P-value for significance. The R statistic typically ranges between 0 and 1, with a higher value indicating a greater degree of distinction among groups. ANOSIM was calculated using the Vegan package for the R software (44). In order to visualize similarities among samples, a non-metric Multidimensional Scaling (nMDS) analysis was employed using the Bray-Curtis dissimilarity matrix. The goal of nMDS is to reduce information from multiple variables into two dimensions using rank orders so that they can be plotted and interpreted in the phase space. All statistical analyses and graphical representations were carried out using the R framework and ggplot2 package (44, 55).

## Results

**Fig. 1:**
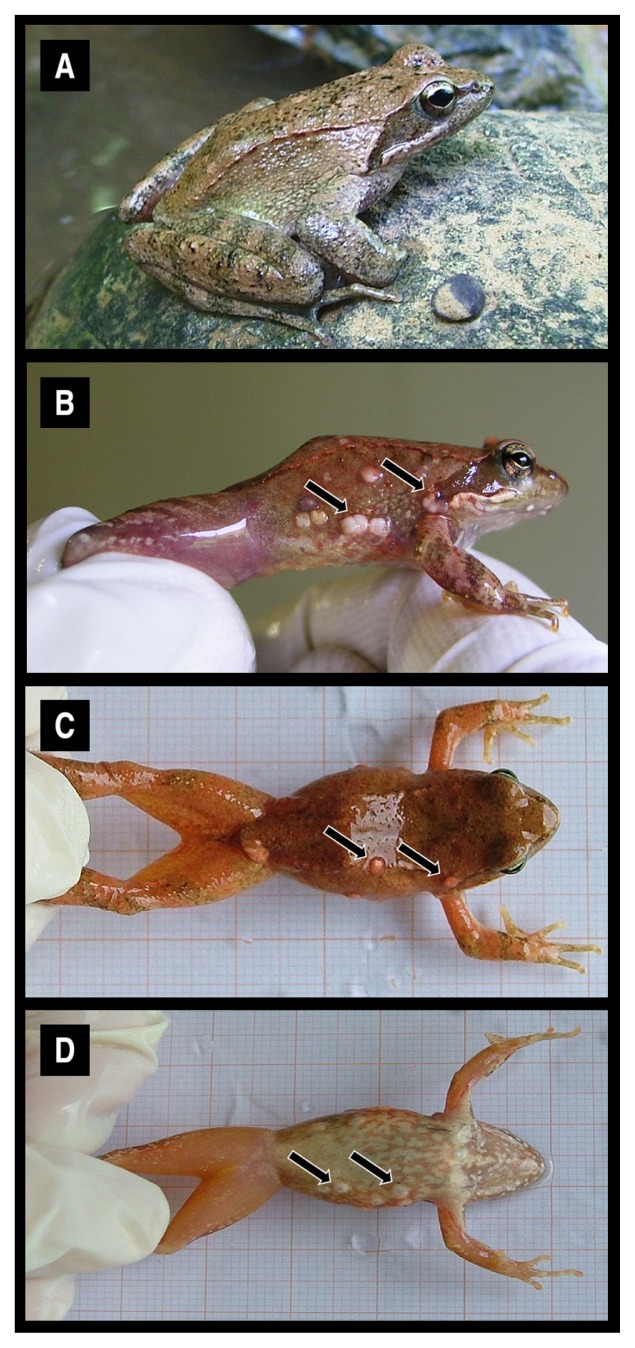
Specimens of Italian stream frogs (*Rana italica*) uninfected (panel A) and infected by a cutaneous parasite of the genus *Amphibiocystidium* (panels B, C, D). Arrows indicate the swellings commonly found on the lateral (B), dorsal (C) and ventral (D) skin of infected individuals.

The *R. italica* specimens collected were divided into two groups: uninfected and infected by parasites of the genus *Amphibiocystidium*. Infected frogs exhibited gross swellings distributed on the dorsal and ventral skin of the head, torso, and limbs (Fig. 1). Further histopathological examinations indicated that the swellings appeared as spherical, ovoid, and/ or U-shape cysts filled with walled endospores. Three representative specimens of each group were selected in order to characterize the skin microbiota.

The total number of microbial OTUs found across the two groups of specimens and calculated applying a threshold of 97% sequence similarity was 717, which was reduced to 629 after removing OTUs with only one sequence read. A total of 127 OTUs were shared by all six specimens. Of these, OTU_001, OTU_002, and OTU_003, identified as belonging to the families *Comamonadaceae*, *Moraxellaceae*, and *Pseudomonadaceae*, respectively, showed abundances ranging from 16.03% to 20.49% and together represented more than 53% of all the shared sequences. Considering OTU_004, OTU_005, and OTU_006, with abundances ranging from 4.31% to 4.74% and belonging to the families *Methylophilaceae*, *Flavobacteriaceae*, and *Oxalobacteraceae*, respectively, these six OTUs represented more than 66% of the common bacterial community.

The OTU richness found across samples, which ranged from 293 to 410, and the other diversity indexes are shown in Table 1. The bacterial diversity indexes were similar in specimens belonging to each group (*i.e.*, uninfected and infected frogs), with uninfected frogs showing a generally higher bacterial diversity than that of infected frogs. The only exception was specimen R154M, an infected frog featuring diversity indexes closer to those obtained in uninfected specimens.

**Fig. 2:**
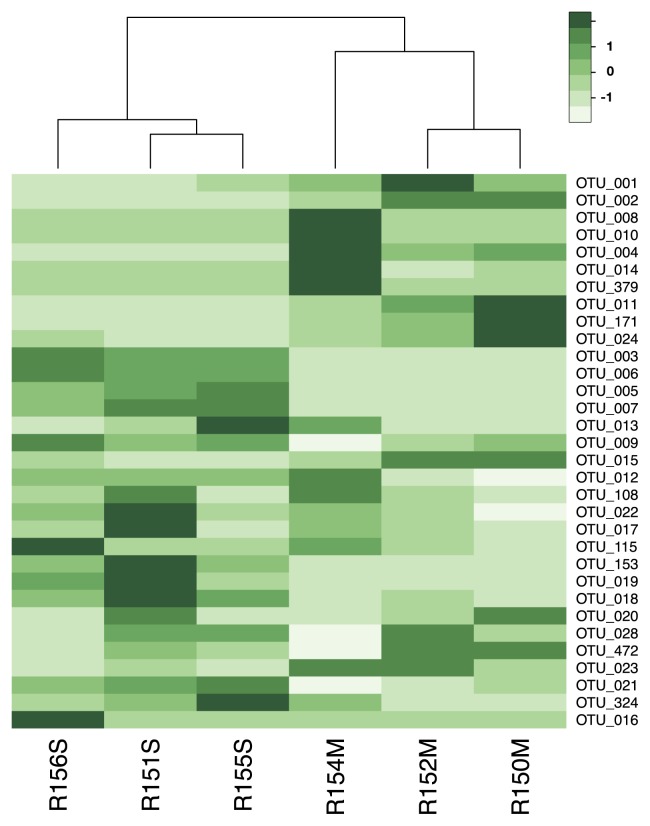
Heatmap showing the relative abundances of the OTUs across specimens uninfected (R151S, R155S, R156S) and infected (R150M, R152M, R154M) by *Amphibiocystidium* and clusters based on Bray-Curtis distances. The OTU abundance cut-off was set to a minimum of 200 reads. Darker green indicates higher values of abundance.

The community structures of the skin microbiota were shown in heatmaps reflecting relative OTU abundances across the six specimens (Fig. 2, a heatmap including only OTUs with a minimum abundance of 200 reads, and Fig. S1, heatmaps with OTU abundance cut-offs ranging from 5 to 600 reads). At all the considered OTU abundance cut-offs, the cluster analysis based on Bray-Curtis distances partitioned the six samples into two main groups, which corresponded to the bacterial communities of uninfected and infected frogs. Furthermore, the distances among uninfected frogs appeared to be shorter than those among infected frogs.

The ANOSIM analysis revealed a slight difference between the two groups (*R*=0.74; *P*=0.091). The nMDS analysis revealed that frogs uninfected by *Amphibiocystidium* had a more distinct bacterial composition than that of the infected frogs (Stress = 0.062) (Fig. S2).

In order to gain further insights into the identity and distribution of the bacterial populations in the skin of uninfected and infected frogs, the filtered and merged sequences obtained from each sample, irrespective of their OTU assignment, were given a taxonomic attribution as described in the Methods section. Excluding phylotypes represented by only one sequence and/or present in only one specimen, we detected a total of 16 different phyla. Of these, the dominant phyla, namely, those with a relative average abundance higher than 1%, were *Proteobacteria* (73.75%), *Bacteroidetes* (11.49%), *Actinobacteria* (4.78%), *Firmicutes* (2.24%), and *Cyanobacteria* (1.39%), which showed similar average abundances in uninfected and infected frogs, with the only exception of *Bacteroidetes*, which was higher in the former (14.86%) than in the latter (8.12%).

**Fig. 3:**
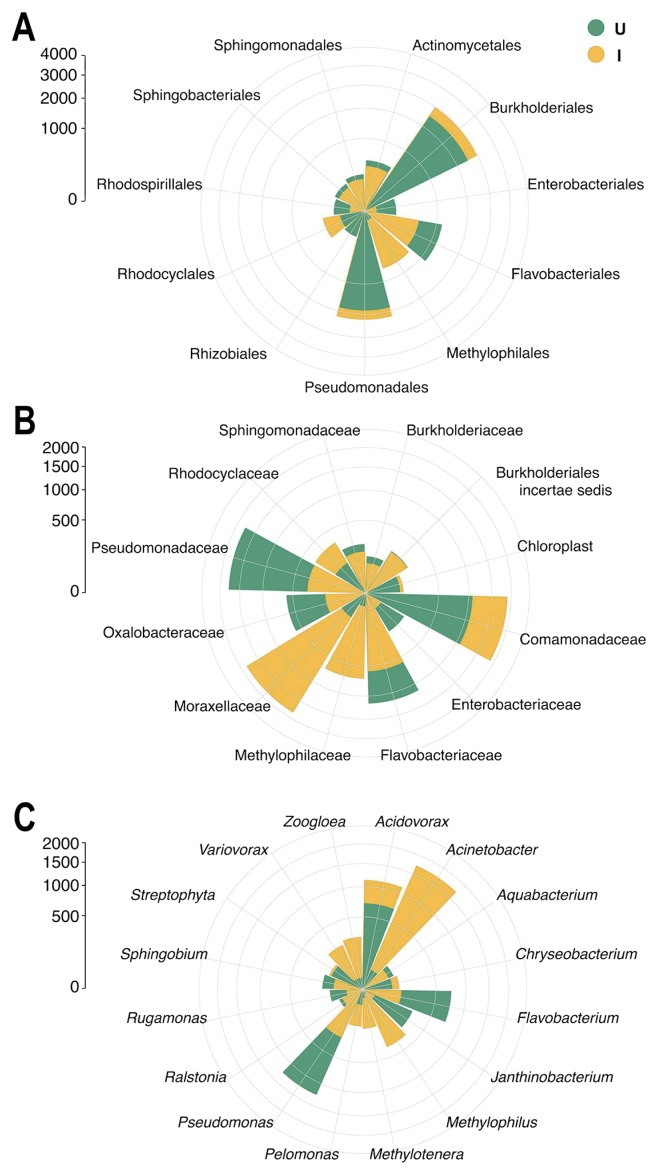
Polar plots showing the average abundances of the most frequent bacterial phylotypes, at the taxonomic levels of order (A), family (B), and genus (C), found on the skin of specimens uninfected (*U*, in green) and infected (*I*, in yellow) by *Amphibiocystidium*.

Among the most abundant orders shown in Fig. 3A, *Burkholderiales*, *Pseudomonadales*, and *Actinomycetales* presented average abundances that were similar in the skin of uninfected and infected frogs (37.81% and 39.49%, 28.15%, and 29.58%, and 7.40% and 5.06%, respectively). In contrast, *Flavobacteriales* were more abundant in uninfected specimens than in infected specimens (17.58% and 7.72%, respectively), while *Methylophilales* was abundant in infected frogs only (9.17%) (Fig. 3A).

The bacterial families *Pseudomonadaceae*, *Flavobacteriaceae*, and *Oxalobacteraceae* were more abundant in the skin of uninfected frogs than in that of infected frogs (31.15% and 5.06%, 20.24% and 8.94%, 10.41% and 2.46%, respectively) (Fig. 3B). *Comamonadaceae*, despite being more prevalent in the infected specimens than in the uninfected specimens (29.23% and 18.98%, respectively), were abundant in both groups (Fig. 3B). Furthermore, bacterial populations belonging to the families *Moraxellaceae* and *Methylophilaceae* were among the most abundant in the infected group of frogs only, in which they showed average abundances of 28.66% and 10.72%, respectively (Fig. 3B).

**Fig. 4:**
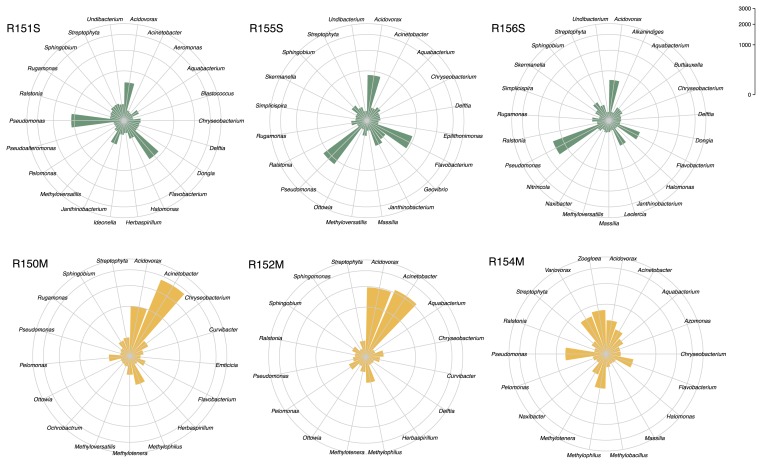
Polar plots showing the abundances of the most frequent bacterial genera, found on the skin of specimens uninfected (R151S, R155S, R156S) and infected (R150M, R152M, R154M) by *Amphibiocystidium*.

The average abundances of the most frequent bacterial genera in the two groups of specimens are shown in Fig. 3C, while the polar plots in Fig. 4 show the most abundant bacterial genera found in each frog specimen. Bacteria belonging to the genus *Acidovorax* showed similar average abundances in uninfected and infected frogs (19.01% and 23.22%, respectively), even though the abundance values were more similar among specimens in the uninfected group (ranging from 16.49% to 21.34%) than those in the infected group (ranging from 10.50% to 37.79%). The genera *Pseudomonas* and *Flavobacterium* showed higher average abundances in uninfected than in infected frogs (33.82% and 5.26%, 20.17% and 2.99%, respectively), and these bacteria within the latter group were abundant in specimen R154M only. Furthermore, the genus *Janthinobacterium* was among the most abundant in uninfected specimens only, with an average of 7.57%. In contrast, bacteria belonging to the genera *Acinetobacter*, *Methylophilus*, and *Methylotenera* were abundant in the infected frogs only, with averages of 35.37%, 7.54%, and 2.95% respectively. Two genera, namely, *Zoogloea* and *Variovorax*, were only abundant in the infected specimen R154M (17.80% and 14.94%, respectively).

## Discussion

Our understanding of the composition and role of the microbiota associated with plants and animals, including humans, is increasing due to the application of high-throughput next-generation sequencing technologies in microbial ecology. Microbial communities living on animal skin represent a particularly interesting scenario as they are continuously exposed to the influence of the external environment. Nevertheless, these studies have largely been limited to the human skin microbiome (39). Among wild animals, amphibians, due to the absence of fur or feathers, provide an excellent model system for studying skin-associated microbial communities, which are considered to mediate susceptibility to diseases because they provide the first line of defense against pathogens (28, 37, 56).

In the present study, we used the Illumina next-generation sequencing of 16S rRNA gene fragments to characterize the skin microbiota of the Italian stream frog (*R. italica*) and investigate its relationship with a cutaneous infectious disease caused by a mesomycetozoean parasite of the genus *Amphibiocystidium*. A similar approach has been used in recent studies to address different aspects of the amphibian skin microbiota, such as the bacterial community composition in co-habiting amphibian species (41), variabilities across species, sampling sites, and developmental stages (35), occurrence in the wild and stability through time in captivity (3, 38), and its relationship with environmental microbes (23, 53). Nevertheless, to the best of our knowledge, this is the first study on the skin microbiota of the Italian stream frog and, in particular, the first to show differences between infected and uninfected frogs with the aim of addressing bacterial populations with the potential to reduce susceptibility to a cutaneous disease in amphibians. Unlike the chytrid fungal skin pathogen *Bd*, it is not possible to culture *Amphibiocystidium* with the aim of carrying out growth inhibition assays (42); therefore, we hypothesized that the identification of protective bacterial taxa may be achieved by comparing the community composition in the skin of uninfected frogs with that of infected frogs. McKenzie and collaborators (41), who studied co-habiting amphibian species, found that one individual of the Western chorus frog (*P. triseriata*) was positive for *Bd*; however, due to the lack of other positive individuals, they were unable to draw any concrete conclusions regarding possible interactions between the infection and skin bacterial community. Previous studies demonstrated that the composition of the skin bacterial community was largely species-specific (41), that a strong developmental shift occurred in skin microbes following metamorphosis (35), and that pond sites represented a secondary influencing factor because, in a host species-specific manner, amphibian skin may select microbes that are generally present in low abundance in the environment (53). Therefore, in the present study, we only considered specimens belonging to the same species and same developmental stage sampled on the same day at the same site, with the aim of isolating the absence/presence of the cutaneous infectious disease from other factors structuring the skin microbiota.

In our survey, we found 629 different OTUs, which showed a per-sample abundance ranging from 293 to 410 and belonging to 16 different phyla. Using a similar approach, Costello *et al.*(14) demonstrated that the human skin microbiome was comprised of 18 different bacterial phyla, while McKenzie *et al.*(41) found between 10 and 18 unique skin bacterial phyla in amphibians, depending on the species. Therefore, the richness of the skin bacterial community that we discovered in the Italian stream frog was similar to that found in humans and other amphibian species. Our results also confirmed the ability, provided by the next-generation sequencing of 16S rRNA genes, to examine the skin bacterial community composition in more depth in respect to other culture-dependent or culture-independent approaches. Woodhams *et al.*(56) cultured 40 unique bacterial isolates belonging to only 3 different phyla from the skin of *Rana muscosa*, while Lauer *et al.*(37) found 19 different bacterial populations, representing only 4 different phyla, in eastern red-backed salamanders (*Plethodon cinereus*) using gradient gel electrophoresis 16S rRNA fingerprinting. We demonstrated that the bacterial community of all specimens was dominated by a limited number of phyla, namely *Proteobacteria*, *Bacteroidetes*, *Actinobacteria*, *Firmicutes*, and *Cyanobacteria*, which were largely the same taxa that were found to be dominant in similar studies (35, 41, 53). Furthermore, in our specimens, the shared bacterial community, namely, the bacterial populations present in the skin of all individuals, was composed of approximately one fifth of all OTUs and dominated by a small number of OTUs. These results are consistent with previous findings obtained using a next-generation sequencing approach, which showed that a few phylotypes dominated the cutaneous bacterial communities of several amphibian species (35, 38, 41, 53).

By comparing frogs uninfected and infected by *Amphibiocystidium* in the present study, a generally higher bacterial diversity was detected in the former. Our results also showed that the two groups had distinct bacterial community structures, as revealed by an nMDS analysis and cluster analysis based on Bray-Curtis distances, indicating that a relationship may exist between the bacterial community and infection occurrence. Furthermore, the shorter distances observed among the uninfected frogs than among the infected frogs indicated that the bacterial communities in the former were more homogenous than in the latter, suggesting the presence of a core microbiota with putative protective activity against the parasitic infection in uninfected frogs. The persistence of a *Bd*-naïve population of *Rana muscosa* was previously associated with a high proportion of individuals with anti-*Bd* bacteria (36). These findings suggested that protection, similar to herd immunity, may be based on a proportion of individuals needing to have anti-*Bd* bacteria in order to protect the population and allow co-existence with the pathogen. Since *Amphibiocystidium* infection was detected in the frog population inhabiting the same collection site of the present study in 2007 (unpublished data), a similar mechanism of protection may also occur against this amphibian skin pathogen. Thus, a closer examination of the composition of bacterial communities, and particularly the identification of bacterial phylotypes with a different distribution between infected and uninfected individuals, may provide an insight into bacterial populations with a protective role.

Most of the bacterial populations detected in the present study belonged to the phylum *Proteobacteria*, and, in particular, the orders *Burkholderiales*, *Pseudomonadales*, and *Methylophilales*. Even if *Burkholderiales* and *Pseudomonadales* showed similar abundances in the skin of uninfected and infected frogs at the order level, their distribution between the two groups varied at lower taxonomic ranks.

Within *Burkholderiales*, bacteria of the family *Oxalobacteraceae* were more abundant in uninfected specimens. Bacteria belonging to the genus *Janthinobacterium* were almost exclusively present in uninfected specimens. *Janthinobacterium* species are among the most extensively studied bacteria with disease-protective roles in amphibians and have been cultured from the skin of various species. *Janthinobacterium* isolates, obtained from *H. scutatum*, showed antifungal activity *in vitro* against *Mariannaea elegans* and *Rhizomucor variabilis* (37), and a strain of *J. lividum*, isolated from the skin of *Plethodon cinereus*, was found to produce antifungal metabolites at concentrations that inhibited the growth of *Bd* (9). *In vivo* studies demonstrated that the addition of *J. lividum* to the skin of *Rana muscosa* prevented morbidity and mortality caused by such a pathogen (29) and also that these effects were, at least in the red-backed salamander (*P. cinereus*), related to increases in the concentration of the antifungal metabolite violacein on the skin (1). Using Illumina technology Loudon *et al.*(38) identified *J. lividum* in a large proportion of red-backed salamanders (*P. cinereus*) both in the wild and captivity. We also showed that another family of *Burkholderiales*, namely, the *Comamonadaceae*, and particularly those of the genus *Acidovorax*, despite being abundant in both groups, prevailed within infected specimens. Members of the family *Comamonadaceae* were previously shown to be abundant in the skin of amphibians, including frogs, salamanders, and newts (35, 38, 41, 43), and strains of *Acidovorax* sp. were isolated from the skin of *Rana cascadae* (50) and *Hemidactylium scutatum* (37).

Within the order *Pseudomonadales*, bacteria belonging to the family *Pseudomonadaceae*, particularly those of the genus *Pseudomonas*, were more abundant in uninfected than infected specimens. Using next-generation sequencing, *Pseudomonadaceae* were found to be among the most abundant phylotypes in frog and salamander species (38, 41). Furthermore, Kueneman *et al.*(35) showed that the occurrence of *Pseudomonas* was high on *R. cascadae* tadpoles and in the water of the lake from which these specimens were captured. Similar findings were reported by Walke *et al.*(53) for juvenile bullfrogs (*Rana catesbeiana*) and adult red-spotted newts (*Notophthalmus viridescens*), as well as the pond substrate inhabited by these individuals. Several *Pseudomonas* species are known to provide protection against pathogenic bacteria and fungi in many plant and animal hosts, and are used as probiotics in agriculture and aquaculture (16, 27, 31). In amphibians, a large number of culture-dependent studies have shown that many pseudomonads may be isolated from the skin of different species and produce antimicrobials capable of inhibiting bacterial and fungal pathogens, including *Bd* (28, 36, 37, 56). Within endangered mountain yellow-legged frogs (*Rana sierrae* and *Rana muscosa*), several *Pseudomonas* species, together with other bacteria capable of inhibiting *Bd* growth *in vitro*, were found to occur more frequently on individuals belonging to populations that co-existed with the pathogen and survived the infection than on those in populations that were extirpated by *Bd* infection (36, 56). Harris *et al.*(30) demonstrated that adding *Pseudomonas reactans* to salamanders (*P. cinereus*) infected with *Bd* ameliorated the effects of chytridiomycosis. We found that, within the *Pseudomonadales*, in contrast to that observed for *Pseudomonadaceae*, bacteria of the family *Moraxellaceae*, particularly those of the genus *Acinetobacter*, were among the most abundant in the infected group of frogs only. Despite *Acinetobacter* species showing anti-*Bd* activity in toads of the genus *Atelopus* (24), these bacteria have also been associated with diseased coral (*Oculina patagonica*) and stress-exposed fish (*Salvelinus fontinalis*) (8, 34). Thus, the occurrence of *Acinetobacter* species in *R. italica* may also be related to stressful conditions, such as the occurrence of *Amphibiocystidium* infection.

We found phylotypes of the order *Methylophilales* almost exclusively on the infected specimens. This order was largely represented by two genera of the family *Methylophilaceae*, namely *Methylophilus* and *Methylotenera*, bacteria commonly found in typical amphibian environments, such as mud as well as river and pond water. *Methylotenera* species were previously detected on Western chorus frogs (41), American bullfrogs (35), and red-spotted newts (53). Nevertheless, to the best of our knowledge, our results on *R. italica* are the first to suggest that these two bacterial genera may be related to stress conditions such as a pathogen infection.

Within the phylum *Proteobacteria*, frog R154M showed, besides those shared with other specimens, further dominant taxa uncharacteristic of its own bacterial community, such as the genera *Zoogloea* and *Variovorax*. Since R154M was the only male frog examined in the present study, we cannot exclude the possibility that its atypical bacterial community may have depended on gender, as previously reported (37).

The second most abundant phylum that we encountered in our survey was represented by *Bacteroidetes*, which was disproportionate in the two groups of specimens, being more abundant in uninfected frogs. This was largely attributed to the very high abundance of phylotypes belonging to the genus *Flavobacterium* (order *Flavobacteriales*, family *Flavobacteriaceae*) in the uninfected specimens. Bacteria belonging to *Flavobacteriaceae* were isolated (43, 50) and identified by next-generation sequencing (3, 35, 41) in the skin of frogs and other amphibians. Furthermore, Lauer *et al.*(37) detected *Flavobacterium* species among the most frequently occurring bacteria, isolated from the skin of salamanders, with antifungal activities, while Lam *et al.*(36) isolated a *Flavobacterium* strain showing anti-*Bd* activity *in vitro* from the skin of *Rana muscosa*. These findings, together with our result showing the abundance of *Flavobacteria* in uninfected specimens of *R. italica*, point toward the possible protective role of these bacteria against cutaneous diseases in amphibians.

## Conclusion

We herein provided, for the first time, evidence for the cutaneous microbiota composition of *R. italica*, which appeared to be dominated by a few OTUs, similar to other amphibian species. By comparing uninfected and infected individuals, we observed a relationship between the structure of the skin bacterial community and occurrence of a cutaneous infectious disease caused by a mesomycetozoean of the genus *Amphibiocystidium*. We demonstrated that the abundances of phylotypes identified as *Pseudomonas*, *Flavobacterium*, and *Janthinobacterium* were higher in uninfected specimens than in infected specimens. Since members of these genera are known to exhibit antifungal activity in amphibians and taking into account the impossibility of culturing *Amphibiocystidium* to perform growth inhibition assays, these results led us to hypothesize that these bacteria may play a role in protecting *R. italica* against the *Amphibiocystidium* infection.

In order to better understand the protective role of skin microorganisms in *R. italica*, it will be necessary to carry out a similar approach in a larger amphibian population and explore further factors affecting the microbiota composition among uninfected and infected hosts, such as different time points, including seasonal cycles and different capture sites. Furthermore, since Kueneman *et al.*(35) demonstrated the occurrence of a marked shift in the skin microbial community following metamorphosis and considering that such a process involves changes in the immune system and in the skin structure that may lead to a greater risk of pathogen infections, it will also be necessary to analyze the microbiome of *R. italica* specimens in different life history stages in order to verify a possible relationship with *Amphibiocystidium* infection.

## Supplementary Information



## Figures and Tables

**Table 1:** Bacterial alpha diversity of individual frogs based on the OTU distribution

Specimen^a^	*S*	*H*	*D*	*J*
R151S (U)	410	3.8	0.93	0.63
R155S (U)	394	3.4	0.90	0.57
R156S (U)	375	3.3	0.87	0.56
R150M (I)	293	2.7	0.79	0.47
R152M (I)	355	2.6	0.78	0.45
R154M (I)	376	3.5	0.92	0.59

a Specimens uninfected and infected by *Amphibiocystidium* are indicated as U and I, respectively.

*S*, OTU richness; *H*, Shannon index; *D*, Simpson index; *J*, Pielou index.
