# Supplementary material for: Characterization of the Skin Microbiota in Italian Stream Frogs (Rana italica) Infected and Uninfected by a Cutaneous Parasitic Disease
Source: Microbes Environ. 2015 Sep 15;30(3):262–9. doi: 10.1264/jsme2.ME15041 (PMC4567565; doi:10.1264/jsme2.ME15041)
Supplement: Supplementary file 1 [file 30_262_s1.pdf]

Fig. S1. Heatmap showing the relative abundances of the OTUs across specimens uninfected (R151S, R155S, R156S) and infected (R150M, R152M, R154M) by Amphibiocystidium and clusters based on Bray-Curtis distances. OTU abundance cut-offs were set to a minimum ranging from 5 to 600 reads). Darker orange indicates higher values of abundance.

Fig. S2. nMDS plot based on Bray-Curtis distances among cutaneous bacterial communities, considered as OTUs, of specimens uninfected (R151S, R155S, R156S) and infected (R150M, R152M, R154M) by Amphibiocystidium.

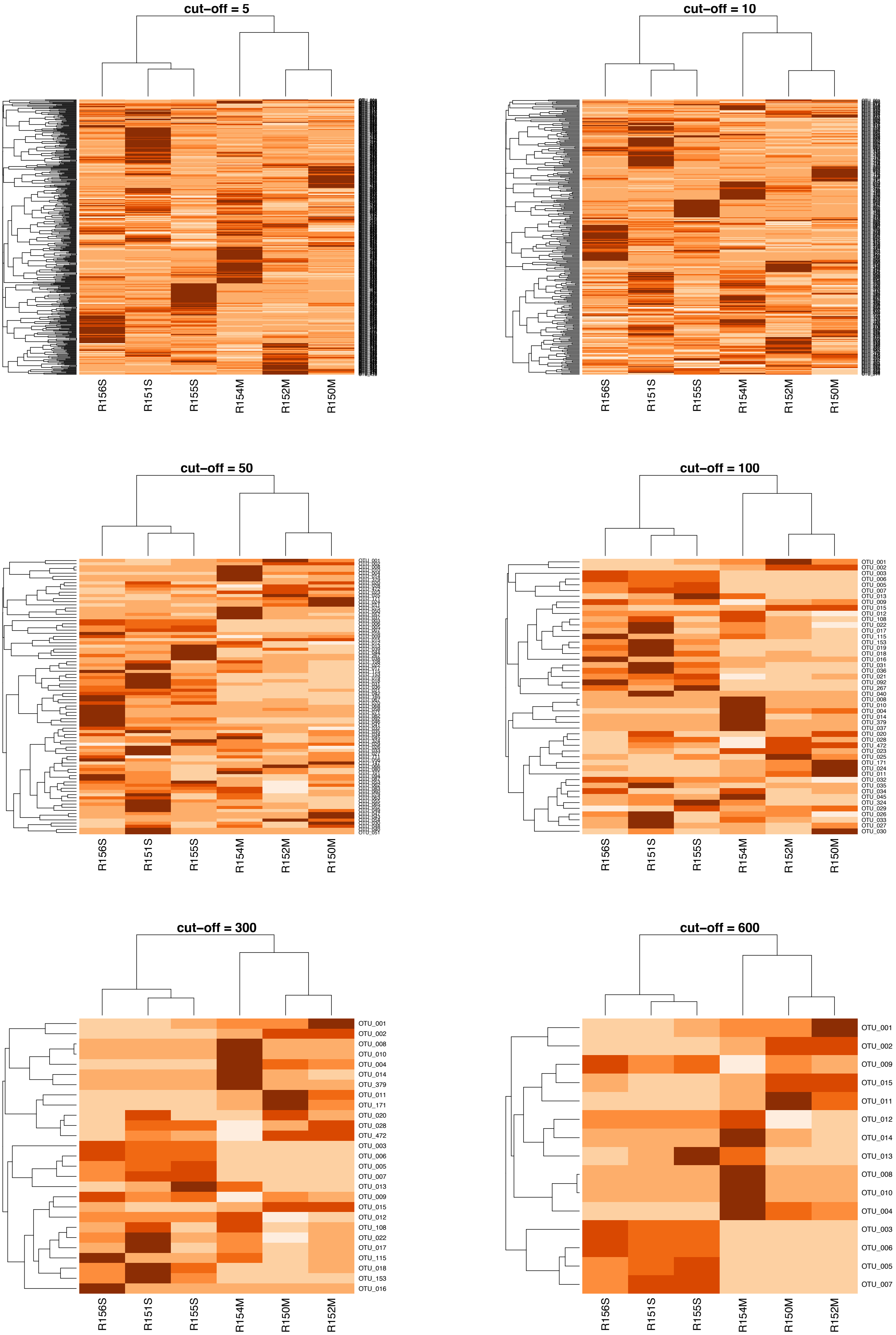
cut–off = 5
cut–off = 10
cut–off = 50
cut–off = 100
cut–off = 300
cut–off = 600
R156S
R151S
R155S
R154M
R152M
R150M
OTU_001
OTU_002
OTU_008
OTU_010
OTU_004
OTU_014
OTU_379
OTU_011
OTU_171
OTU_020
OTU_028
OTU_472
OTU_003
OTU_006
OTU_005
OTU_007
OTU_013
OTU_009
OTU_015
OTU_012
OTU_108
OTU_022
OTU_017
OTU_115
OTU_018
OTU_153
OTU_016

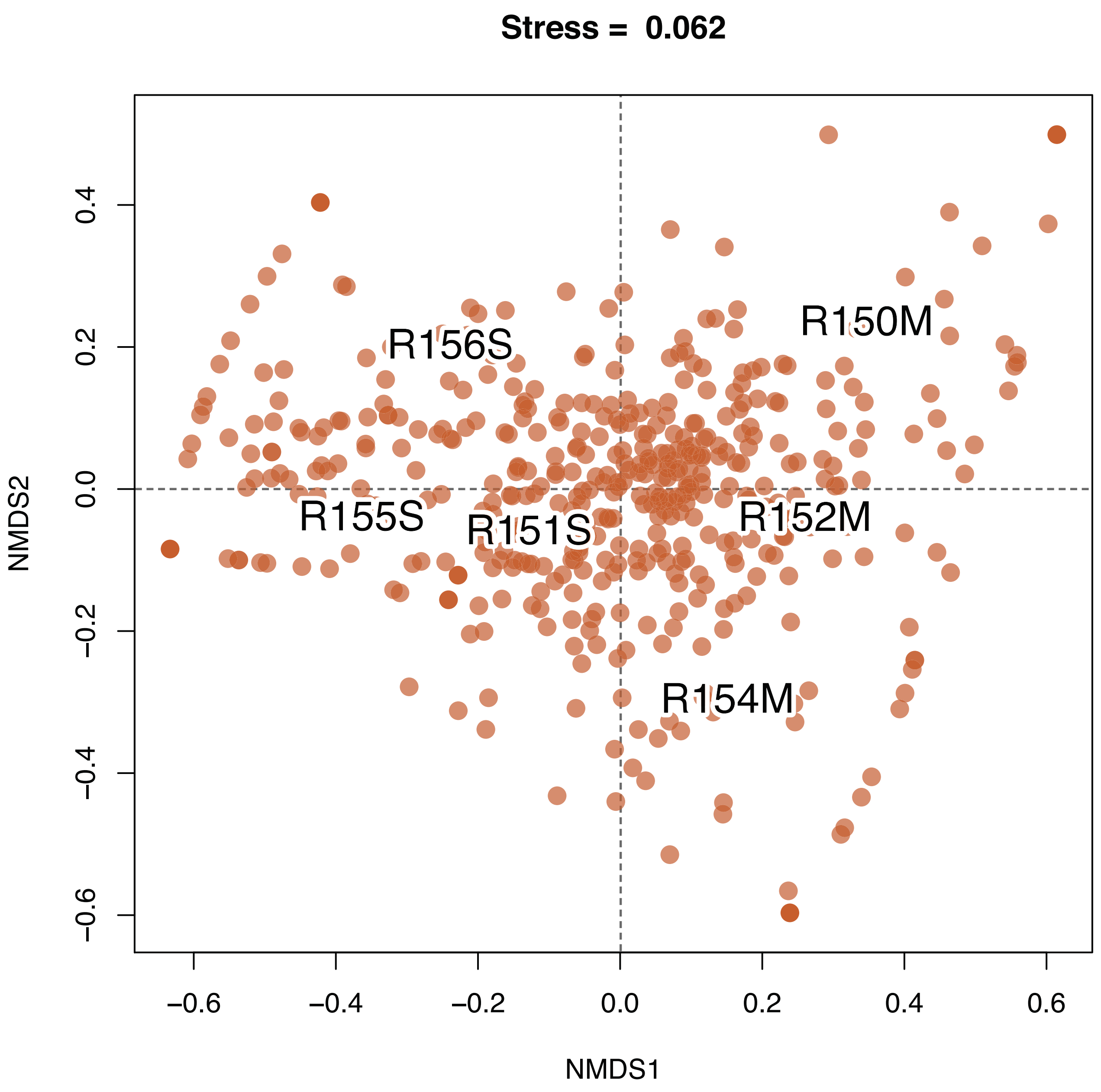
Stress = 0.062
R150M
R156S
R155S
R151S
R152M
R154M
NMDS1
NMDS2
−0.6
−0.4
−0.2
0.0
0.2
0.4
0.6
